# Association of the Frailty-to-Estimated Cardiorespiratory Fitness Ratio with Prevalent Stroke in Middle-Aged and Older Adults: A Cross-Sectional NHANES Study

**DOI:** 10.3390/bioengineering13070750

**Published:** 2026-06-26

**Authors:** Yingchao He, Wendi Yuan, Mingfeng Lv, Yi Yang, Yiya Xu, Zhiwei Song, Xiaolin Jiang, Lvqing Yang, Chenxi Huang, Ying Chen, Yinzhou Wang

**Affiliations:** 1 Department of Neurology, Fuzhou University Affiliated Provincial Hospital, Gulou District, Fuzhou 350000, China; faithcc@fjmu.edu.cn (Y.H.); xiaolinjiang_lish@fjmu.edu.cn (X.J.); 2 Shengli Clinical Medical College of Fujian Medical University, No. 88, Traffic Road, Gulou District, Fuzhou 350000, China; xuyy@fjmu.edu.cn; 3 Institute of Artificial Intelligence, Xiamen University, No. 4221, Xiang’an South Road, Xiang’an District, Xiamen 361000, China; windyuan@stu.xmu.edu.cn (W.Y.); young1@stu.xmu.edu.cn (Y.Y.); 4 School of Informatics, Xiamen University, No. 4221, Xiang’an South Road, Xiang’an District, Xiamen 361000, China; lvmingfeng@stu.xmu.edu.cn (M.L.); lqyang@xmu.edu.cn (L.Y.); supermonkeyxi@xmu.edu.cn (C.H.); 5 Fujian Key Laboratory of Medical Analysis, Fujian Academy of Medical Sciences, No. 7, Wusi Road, Gulou District, Fuzhou 350000, China; songzhiwei@fjmu.edu.cn

**Keywords:** prevalent stroke, Frailty Index, estimated cardiorespiratory fitness, Frailty-to-estimated Cardiorespiratory Fitness Ratio, NHANES, cross-sectional study

## Abstract

Stroke remains a major cause of disability worldwide, and population-level indicators that integrate multidimensional vulnerability with physiological reserve may provide useful perspectives for characterizing cerebrovascular health in aging populations. This cross-sectional study examined the association between the Frailty-to-estimated Cardiorespiratory Fitness Ratio (FCR) and self-reported prevalent stroke among middle-aged and older adults using NHANES 2011–2014 data. FCR was calculated as a 23-item modified Frailty Index divided by estimated cardiorespiratory fitness, providing an interpretable load-to-reserve measure that linked accumulated health deficits with estimated cardiorespiratory reserve. The final analytical sample included 3511 participants aged 45 years or older. Survey-weighted logistic regression models, sensitivity analyses, exploratory subgroup analyses, and spline models were used to evaluate the association. The weighted prevalence of self-reported prevalent stroke increased across FCR quartiles, from 2.2% in Q1 to 12.9% in Q4. In the conventional clinical adjustment model, participants in the highest FCR quartile had greater odds of self-reported prevalent stroke than those in the lowest quartile (OR = 6.13, 95% CI: 2.97–12.66; p<0.001), with similar findings in the overlap-aware model (OR = 6.31, 95% CI: 3.07–12.97; p<0.001). Log-transformed FCR was also consistently associated with greater odds of self-reported prevalent stroke across adjustment models, and spline analysis suggested a generally increasing association. These findings support FCR, particularly when modeled using quartiles or log-transformed values, as an interpretable integrative load-to-reserve construct associated with self-reported prevalent stroke, and suggest its potential relevance for population-based characterization of cerebrovascular health in aging adults.

## 1. Introduction

Stroke, characterized as a central nervous system disorder arising from vascular pathology, is clinically classified into ischemic and hemorrhagic subtypes. It remains a critical global health challenge, currently ranking as the second leading cause of mortality and the primary cause of long-term disability in adults worldwide [1]. According to recent data from the Global Burden of Disease (GBD) study, the global burden of stroke has increased substantially since 1990. By 2021, the aggregate burden reached 160 million Disability-Adjusted Life Years (DALYs), with a prevalence of 93.8 million cases and 7.3 million deaths, representing a substantial challenge for public health systems globally [2]. This burden is particularly acute in China, where stroke has become one of the leading causes of death and disability. Statistics from 2020 indicate a prevalence of approximately 17.8 million cases, alongside 3.4 million new cases and 2.3 million stroke-related deaths annually [3,4]. As the global population ages, the incidence and mortality of cardiovascular diseases, including stroke, continue to rise among older adults. This epidemiological context highlights the need to better characterize population-level health indicators associated with stroke burden, particularly those reflecting multidimensional vulnerability and physiological reserve [5,6].

To better capture heterogeneity in health status among aging populations, the Frailty Index (FI) has been widely used to quantify biological vulnerability beyond chronological age. Based on the deficit accumulation framework, the FI summarizes the proportion of health deficits present across multiple domains, including chronic conditions, functional limitations, self-rated health, and healthcare use [7,8,9]. This accumulated deficit burden reflects reduced physiological reserve and increased vulnerability to stressors, helping to explain why individuals of similar chronological age may differ substantially in health status and clinical outcomes. Previous studies have linked higher FI levels with mortality, cardiovascular risk factors, cardiovascular disease, heart failure, and stroke-related outcomes [6,10,11,12,13,14,15,16]. Moreover, frailty is potentially dynamic, and changes in frailty status have been associated with subsequent cardiovascular outcomes in older adults [17]. These findings suggest that FI is a useful population-level indicator of multidimensional vulnerability in studies of cerebrovascular health.

While FI reflects accumulated deficit burden, cardiorespiratory fitness (CRF) represents the integrated functional capacity of the respiratory, cardiovascular, and musculoskeletal systems during sustained physical exertion [18]. Higher CRF has been consistently associated with lower all-cause mortality and cardiovascular morbidity [19]. Although maximal oxygen uptake ($VO_{2max}$) measured by cardiopulmonary exercise testing remains the reference standard for CRF assessment, its broad implementation in large epidemiological or routine clinical settings is limited by cost, specialized equipment, and logistical complexity [20]. Consequently, non-exercise estimated cardiorespiratory fitness (eCRF), derived from routinely available demographic, clinical, and lifestyle variables, has been proposed as a practical proxy for CRF. Previous studies have shown that eCRF is associated with directly measured fitness and with mortality and cardiovascular outcomes, supporting its use in population-based research when measured CRF is unavailable [21,22].

Although FI and eCRF are each relevant to cardiovascular and cerebrovascular health, they represent complementary dimensions of aging-related health status: FI reflects accumulated deficit burden, whereas eCRF reflects estimated cardiorespiratory reserve. Considering either dimension alone may emphasize only one aspect of multidimensional health vulnerability. For example, some individuals may have relatively low deficit burden but poor estimated fitness, whereas others may maintain higher estimated fitness despite multiple chronic conditions. To summarize this balance, we constructed the Frailty-to-estimated Cardiorespiratory Fitness Ratio (FCR), calculated as FI divided by eCRF, as an exploratory ratio-based load-to-reserve indicator. We examined whether higher FCR was associated with greater odds of self-reported prevalent stroke among middle-aged and older adults using NHANES 2011–2014 data. We further evaluated the robustness of this association across survey-weighted models and sensitivity analyses, and contextualized FCR relative to FI, eCRF, and their joint model.

## 2. Materials and Methods

### 2.1. Study Design and Population

This cross-sectional study examined the association between the Frailty-to-estimated Cardiorespiratory Fitness Ratio (FCR) and self-reported prevalent stroke among middle-aged and older adults. Data were obtained from the National Health and Nutrition Examination Survey (NHANES), a nationally representative survey of the civilian non-institutionalized U.S. population conducted by the National Center for Health Statistics (NCHS). NHANES uses a complex, stratified, multistage probability sampling design and collects data through standardized in-home interviews, physical examinations, and laboratory assessments conducted in mobile examination centers.

**Figure 1:**
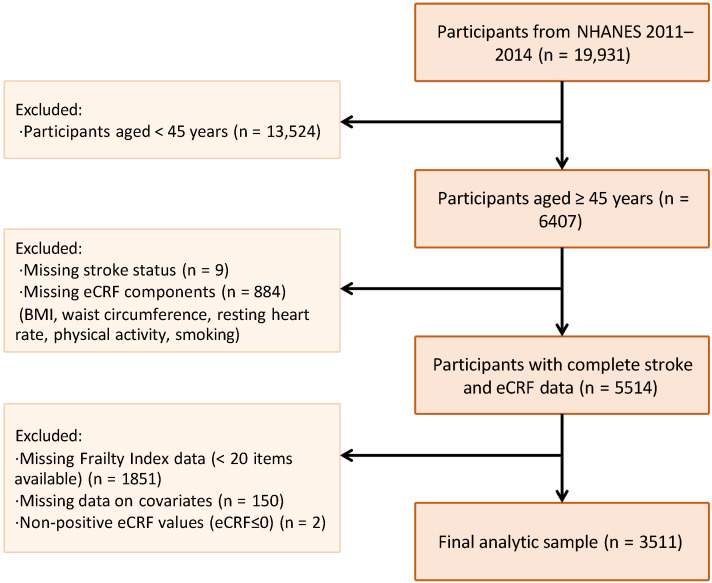
Participant selection flowchart for NHANES 2011–2014.

For the present analysis, we combined two continuous NHANES cycles, 2011–2012 and 2013–2014. The initial dataset included 19,931 participants. As shown in Figure 1, participants younger than 45 years were excluded (n= 13,524), leaving 6407 middle-aged and older adults. We then excluded participants with missing self-reported stroke status (n=9), resulting in 6398 participants with non-missing stroke information. Participants with incomplete components required for eCRF calculation were further excluded (n=884), leaving 5514 participants with complete eCRF data. Among these participants, 1851 were excluded because they had fewer than 20 valid FI items, resulting in 3663 participants with a valid FI. We then excluded participants with missing model covariates or survey design variables (n=150), leaving 3513 complete-case participants before the eCRF positivity check. Finally, because FCR is a ratio-based measure and requires a positive denominator, participants with non-positive raw eCRF values were excluded (n=2). The final analytical sample included 3511 participants.

Participants were categorized into quartiles according to the distribution of FCR in the final analytical sample for descriptive and regression analyses. The NHANES examination weights, strata, and primary sampling units were incorporated in survey-weighted analyses, as described below.

The NHANES protocol was approved by the NCHS Research Ethics Review Board, and all participants provided written informed consent. Because this study used publicly available de-identified NHANES data, no additional institutional review board approval was required.

### 2.2. Outcome Assessment

The outcome of this study was self-reported prevalent stroke. Stroke status was ascertained using the NHANES Medical Conditions Questionnaire (MCQ), in which participants were asked the standardized question, “Has a doctor or other health professional ever told you that you had a stroke?” Participants who answered “Yes” were classified as having self-reported prevalent stroke. NHANES does not provide information on stroke onset date, subtype, severity, or adjudicated clinical endpoints for this analysis; therefore, the outcome represents self-reported prevalent stroke.

### 2.3. Assessment of Key Indicators

Estimated cardiorespiratory fitness (eCRF) was calculated using the sex-specific non-exercise Jackson algorithm [23,24]. This algorithm estimates maximal oxygen uptake ($VO_{2max}$), expressed in metabolic equivalents (METs), using routinely available demographic, anthropometric, physiological, behavioral, and lifestyle variables. The variables included age, body mass index (BMI), waist circumference (WC), resting heart rate (RHR), self-reported physical activity (PA), and smoking status. Because direct cardiopulmonary exercise testing was not available in NHANES, eCRF was used as an estimated proxy for CRF in this population-based analysis. The sex-specific eCRF equations were as follows:

For men:

(1) $$\begin{array}{cc} eCRF= & 21.2870+\left(0.1654\times Age\right)-\left(0.0023\times Age^{2}\right)-\left(0.2318\times BMI\right)\\ \; & -(0.0337\times WC)-(0.0390\times RHR)+(0.6351\times PA)\\ \; & -(0.4263\times Smoke) \end{array}$$

For women:

(2) $$\begin{array}{cc} eCRF= & 14.7873+\left(0.1159\times Age\right)-\left(0.0017\times Age^{2}\right)-\left(0.1534\times BMI\right)\\ \; & -(0.0088\times WC)-(0.0364\times RHR)+(0.5987\times PA)\\ \; & -(0.2994\times Smoke) \end{array}$$

In these equations, PA was coded as 1 for physically active participants and 0 for inactive participants, and Smoke was coded as 1 for current smokers and 0 otherwise.

A 23-item modified Frailty Index (FI) was constructed based on the deficit accumulation framework using health deficits available in NHANES 2011–2014. To avoid direct circularity with the study outcome, self-reported stroke was not included as an FI item. The selected deficits covered four domains: chronic conditions, functional limitations, healthcare use, and self-rated health. Each deficit was coded as 0 for absence and 1 for presence. Participants were required to have at least 20 valid FI items, corresponding to at least 80% item availability. For each participant, the FI was calculated as the number of deficits present divided by the number of valid FI items. Detailed information on the 23 FI items, NHANES variable codes, coding rules, missing proportions, and deficit prevalence is provided in Supplementary Table S1. Given that the modified FI was constructed from available NHANES variables, it was interpreted as an operational deficit-accumulation measure, and alternative FI specifications were examined in sensitivity analyses to assess the influence of selected item domains.

The Frailty-to-estimated Cardiorespiratory Fitness Ratio (FCR) was calculated as FI divided by eCRF:

(3) $$FCR=\frac{FI}{eCRF}$$

FCR was treated as an exploratory ratio-based load-to-reserve indicator, with FI representing accumulated deficit burden and eCRF representing estimated cardiorespiratory reserve. Because ratio variables may be sensitive to small denominator values, the main analysis excluded participants with non-positive raw eCRF values. In addition, log-transformed FCR, FCR quartiles, and extreme-value sensitivity analyses were used to evaluate the robustness of the association.

### 2.4. Statistical Analysis

All analyses incorporated the complex sampling design of NHANES. Because two 2-year NHANES cycles were combined, 4-year MEC examination weights were calculated by dividing the 2-year MEC weights by two. Survey design objects incorporated primary sampling units (SDMVPSU), strata (SDMVSTRA), and 4-year MEC weights, with nesting specified.

Baseline characteristics were summarized across FCR quartiles. Continuous variables were presented as survey-weighted means with standard errors (SEs), and categorical variables were presented as unweighted counts with survey-weighted percentages. Differences across FCR quartiles were assessed using design-based tests, including survey-weighted linear regression or Wald tests for continuous variables and Rao–Scott chi-square tests for categorical variables.

Survey-weighted logistic regression models were used to estimate odds ratios (ORs) and 95% confidence intervals (CIs) for the association between FCR and self-reported prevalent stroke. In the main analyses, FCR was modeled using standardized log-transformed values, defined as log(FCR+0.001) per 1-SD increment, and as quartiles, with the lowest quartile as the reference group. Raw standardized FCR was evaluated in sensitivity analyses because FCR is a right-skewed ratio-based variable. Linear trends across quartiles were tested by entering the median value of each FCR quartile as a continuous term.

Five model specifications were evaluated. Model 1 was unadjusted. Model 2 adjusted for age, sex, and race/ethnicity. Model 3 additionally adjusted for education level, body mass index, smoking status, and alcohol consumption. Model 4 was specified as a conventional clinical adjustment model that further included hypertension and diabetes. Given the partial conceptual overlap between several conventional covariates and the construction of FI or eCRF, we additionally fitted Model 5 as an overlap-aware model adjusted for race/ethnicity, education level, and alcohol consumption. This model was used to evaluate whether the association was consistent under a less overlapping covariate structure.

Because FCR is a ratio-based measure and may be sensitive to small denominator values and extreme ratio values, additional sensitivity analyses were performed. These analyses evaluated log-transformed FCR, defined as log(FCR+0.001), FCR quartiles, the standardized difference between FI and eCRF ($FI_{z}-eCRF_{z}$), exclusion of participants with raw eCRF <1, winsorization of FCR at the 99th percentile, exclusion of the top 1% of FCR values, and a legacy floor approach in which eCRF values <1 were set to 1. These analyses were used to assess the stability of the association under alternative treatments of low eCRF and extreme FCR values. Detailed specifications and results of these sensitivity analyses are provided in Supplementary Table S2.

To benchmark FCR against its individual components, exploratory component-comparison analyses were performed. A baseline covariate model including age, sex, race/ethnicity, education level, and alcohol consumption was compared with models additionally including FI, eCRF, FI plus eCRF, raw FCR, log-transformed FCR, or FCR quartiles. Apparent model performance was assessed using survey-weighted area under the receiver operating characteristic curve (AUC) and related performance metrics, including change in AUC compared with the baseline model, Brier score, calibration indices, discrimination slope, integrated discrimination improvement, and continuous net reclassification improvement. These analyses were used to contextualize the FCR construct relative to FI and eCRF and to describe apparent model performance. Detailed apparent model-performance metrics are provided in Supplementary Table S3A,B.

To assess potential variable overlap and multicollinearity, Spearman correlation coefficients and variance inflation factors were calculated for FCR, FI, eCRF, and selected covariates that overlapped with FI or eCRF construction. Additional overlap-aware adjustment models were fitted, including an overlap-aware model and an overlap-aware model retaining major clinical comorbidities. These analyses were used to evaluate the robustness of the association across alternative covariate adjustment strategies. Correlation and multicollinearity diagnostics are shown in Supplementary Table S4A,B, and results from overlap-aware and alternative adjustment models are provided in Supplementary Table S5.

Exploratory subgroup analyses were conducted using standardized log-transformed FCR across strata defined by age, sex, race/ethnicity, education level, obesity status, hypertension, diabetes, and alcohol use. Survey-weighted logistic regression models were fitted within each subgroup using the overlap-aware adjustment framework where applicable. Interaction terms between log-transformed FCR and subgroup variables were tested, and subgroup findings were interpreted as hypothesis-generating.

To examine the shape of the association, survey-weighted natural spline models were fitted using standardized log-transformed FCR. The overall spline association and evidence of nonlinearity were evaluated using design-based Wald tests. The spline analysis was used to visualize the exposure–outcome relationship and to assess whether the association departed from a linear pattern.

To evaluate potential selection related to missingness, baseline characteristics were compared between participants included in the final analytical sample and eligible participants excluded because of incomplete eCRF components, invalid FI, missing covariates or survey design variables, or non-positive eCRF values. Additional sensitivity analyses were conducted using alternative FI specifications that excluded selected item domains, including hypertension/diabetes, healthcare use, and functional limitation items. Characteristics of included and excluded eligible participants are shown in Supplementary Table S6, and detailed results of the alternative FI specification analyses are provided in Supplementary Table S7.

All statistical tests were two-sided, and p<0.05 was considered statistically significant. Subgroup, sensitivity, and apparent model-performance analyses were considered exploratory. Analyses were conducted using R software version 4.5.2 (R Foundation for Statistical Computing, Vienna, Austria).

## 3. Results

### 3.1. Baseline Characteristics

The final analytical sample included 3511 participants from the NHANES 2011–2014 cycles. The survey-weighted mean age was 65.58 years (SE, 0.20), and 1698 participants were male, corresponding to a weighted percentage of 46.1%. Detailed baseline characteristics according to FCR quartiles are summarized in Table 1. Most demographic, behavioral, clinical, and index-related characteristics differed significantly across FCR quartiles, whereas race/ethnicity did not differ significantly.

Compared with participants in the lowest FCR quartile, those in the highest FCR quartile were older and more frequently female. The weighted mean age increased from 63.51 years in Q1 to 66.64 years in Q4, and the weighted proportion of women increased from 40.0% to 72.7%. Participants in higher FCR quartiles also had lower educational attainment and a less favorable cardiometabolic profile, including higher body mass index, larger waist circumference, and higher prevalence of current smoking, hypertension, and diabetes. For example, from Q1 to Q4, weighted mean BMI increased from 26.41 to 34.18 kg/m^2^, waist circumference increased from 96.65 to 112.50 cm, hypertension prevalence increased from 26.2% to 80.6%, and diabetes prevalence increased from 3.8% to 38.9%.

Consistent with the definition of FCR, higher FCR quartiles were characterized by progressively higher FI and lower eCRF. The survey-weighted mean FI increased from 0.07 in Q1 to 0.43 in Q4, whereas mean eCRF decreased from 9.40 to 6.12 METs. The weighted prevalence of self-reported prevalent stroke also increased across FCR quartiles, from 2.2% in Q1 to 12.9% in Q4.

### 3.2. Association Between FCR and Self-Reported Prevalent Stroke

Survey-weighted logistic regression models were used to examine the association between FCR and self-reported prevalent stroke. Table 2 summarizes the ORs and 95% CIs for log-transformed FCR and FCR quartiles across the prespecified adjustment models. Accordingly, the main regression results are interpreted primarily for log-transformed FCR and quartile-based FCR, whereas raw continuous FCR is described below as a sensitivity parameterization.

The adjustment models were defined as follows: Model 1 was unadjusted; Model 2 adjusted for age, sex, and race/ethnicity; Model 3 additionally adjusted for education level, body mass index, smoking status, and alcohol consumption; Model 4 additionally included hypertension and diabetes as a conventional clinical adjustment model; and Model 5 was an overlap-aware reduced adjustment model including race/ethnicity, education level, and alcohol consumption.

When FCR was modeled in quartiles, higher FCR categories, especially Q3 and Q4, were associated with greater odds of self-reported prevalent stroke. In the unadjusted model (Model 1), compared with participants in Q1, the ORs were 1.76 (95% CI: 0.72–4.27; p=0.222) for Q2, 3.26 (95% CI: 1.63–6.54; p=0.002) for Q3, and 6.67 (95% CI: 3.25–13.69; p<0.001) for Q4, with a significant trend across quartiles (*p* for trend <0.001). The association for the highest quartile remained evident after demographic adjustment in Model 2 (Q4 vs. Q1: OR = 7.02, 95% CI: 3.65–13.48; p<0.001) and after additional adjustment for socioeconomic and lifestyle factors in Model 3 (Q4 vs. Q1: OR = 8.67, 95% CI: 4.19–17.94; p<0.001).

In the conventional clinical adjustment model (Model 4), higher FCR quartiles remained associated with greater odds of self-reported prevalent stroke, particularly for Q3 and Q4. Compared with Q1, the OR was 1.49 (95% CI: 0.60–3.71; p=0.407) for Q2, 2.57 (95% CI: 1.30–5.09; p=0.016) for Q3, and 6.13 (95% CI: 2.97–12.66; p<0.001) for Q4, with a significant trend across quartiles (*p* for trend <0.001). Similar results were observed in the overlap-aware model (Model 5), where the ORs were 1.77 (95% CI: 0.74–4.27; p=0.215) for Q2, 3.15 (95% CI: 1.57–6.34; p=0.004) for Q3, and 6.31 (95% CI: 3.07–12.97; p<0.001) for Q4. These findings support a consistent quartile-based association between higher FCR and self-reported prevalent stroke across conventional and overlap-aware adjustment frameworks.

When FCR was modeled as a raw standardized continuous variable, the estimates were attenuated in the conventional clinical adjustment model (OR = 1.06, 95% CI: 0.94–1.18; p=0.367) and in the overlap-aware model (OR = 1.10, 95% CI: 0.58–2.07; p=0.775). Therefore, subsequent sensitivity analyses further evaluated log-transformed FCR and quartile-based FCR to address the ratio-based distribution and potential influence of extreme FCR values.

### 3.3. Sensitivity and Component-Comparison Analyses

Given the ratio-based nature of FCR, additional sensitivity analyses were conducted to evaluate the influence of low eCRF values and extreme FCR distributions. Raw continuous FCR showed attenuated associations, whereas log-transformed FCR was positively associated with self-reported prevalent stroke (OR per 1-SD increment = 2.25, 95% CI: 1.83–2.76; p<0.001). The alternative standardized composite $FI_{z}-eCRF_{z}$ also showed a positive association (OR per 1-SD increment = 1.90, 95% CI: 1.61–2.25; p<0.001). Similar patterns were observed in analyses based on FCR quartiles and in sensitivity analyses addressing extreme FCR values, supporting the use of log-transformed and categorical FCR representations. Detailed sensitivity results for FCR parameterization and extreme values are provided in Supplementary Table S2, and alternative FI specification analyses are provided in Supplementary Table S7.

In exploratory model-comparison analyses, log-transformed FCR and FCR quartiles improved apparent discrimination compared with the baseline covariate model (AUC: 0.7305 and 0.7205 vs. 0.6423, respectively). FI-based models showed numerically higher apparent AUCs (FI alone: 0.7485; FI plus eCRF: 0.7495). These results suggest that FCR, particularly in log-transformed or quartile-based form, may provide a complementary and interpretable load-to-reserve framework rather than a replacement for FI or eCRF considered separately.

### 3.4. Exploratory Subgroup Analyses

**Figure 2:**
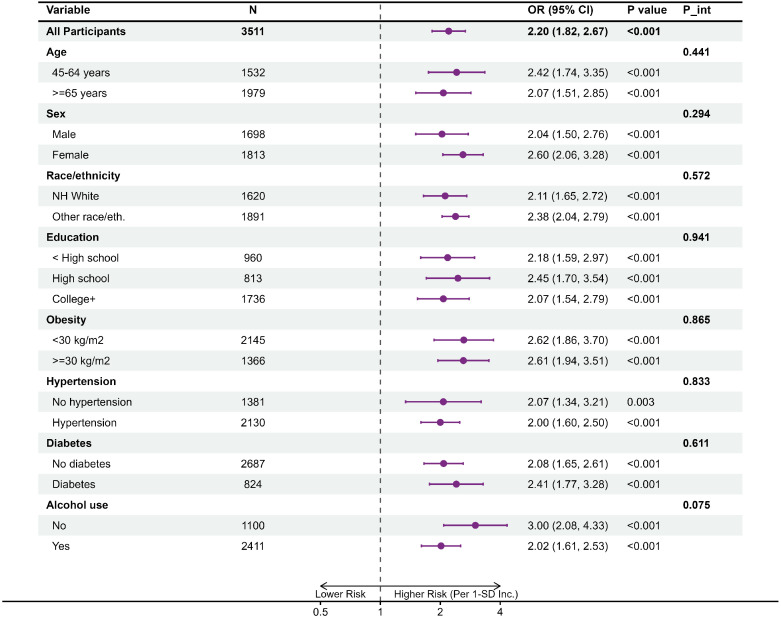
Subgroup associations of standardized log(FCR+0.001) with self-reported prevalent stroke. ORs are shown per 1-SD increment.

Exploratory survey-weighted subgroup analyses were performed to examine whether the association between standardized log-transformed FCR and self-reported prevalent stroke differed across prespecified strata (Figure 2). Overall, the estimated associations were directionally similar across the examined subgroups, including strata defined by age, sex, race/ethnicity, education level, obesity status, hypertension, diabetes, and alcohol use.

The point estimates were directionally consistent across subgroups. For example, the ORs per 1-SD increment in log(FCR+0.001) were 2.42 (95% CI: 1.74–3.35) among participants aged 45–64 years and 2.07 (95% CI: 1.51–2.85) among those aged ≥65 years. The corresponding ORs were 2.04 (95% CI: 1.50–2.76) in men and 2.60 (95% CI: 2.06–3.28) in women; 2.62 (95% CI: 1.86–3.70) in participants with BMI <30 kg/m^2^ and 2.61 (95% CI: 1.94–3.51) in those with BMI ≥30 kg/m^2^; and 2.07 (95% CI: 1.34–3.21) among participants without hypertension and 2.00 (95% CI: 1.60–2.50) among those with hypertension.

No statistically significant interactions were observed for age, sex, race/ethnicity, education level, obesity status, hypertension, diabetes, or alcohol use (all *p* for interaction >0.05). These findings suggest that the association between log-transformed FCR and self-reported prevalent stroke was broadly similar across prespecified subgroups; however, the subgroup analyses should be interpreted as exploratory and hypothesis-generating.

### 3.5. Spline Analysis of Log-Transformed FCR

**Figure 3:**
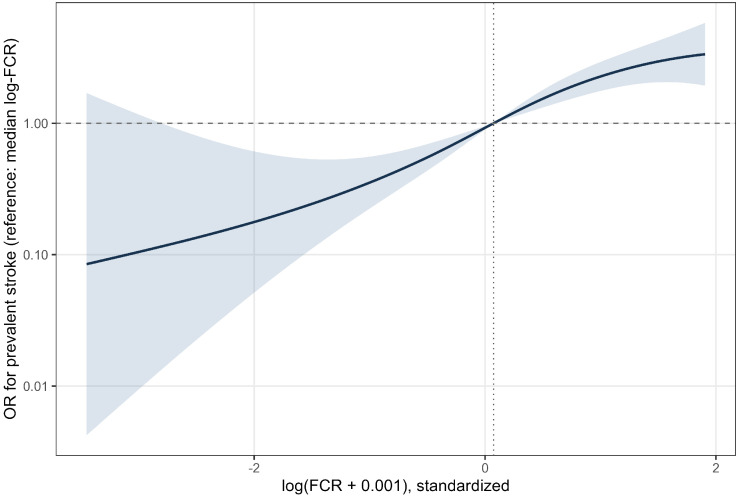
Survey-weighted spline association between standardized log(FCR+0.001) and self-reported prevalent stroke. ORs are referenced to the median value; shading indicates 95% CIs; reference lines indicate OR = 1 and the median value.

To visualize the shape of the association between log-transformed FCR and self-reported prevalent stroke, we fitted survey-weighted spline models using standardized log-transformed FCR, defined as log(FCR+0.001). As shown in Figure 3, the estimated odds of self-reported prevalent stroke tended to increase with higher log-transformed FCR. The overall spline association was statistically significant (p<0.001), whereas evidence for nonlinearity was not statistically significant (*p* for nonlinearity = 0.222). These findings suggest a generally increasing association between log-transformed FCR and self-reported prevalent stroke.

## 4. Discussion

In this cross-sectional analysis of middle-aged and older adults from NHANES 2011–2014, higher FCR was associated with greater odds of self-reported prevalent stroke. The association was most evident when FCR was modeled using quartiles or log-transformed continuous values. In survey-weighted logistic regression models, participants in the highest FCR quartile had substantially higher odds of self-reported prevalent stroke than those in the lowest quartile, both in the conventional clinical adjustment model and in the overlap-aware model. The spline analysis further suggested a generally increasing association between log-transformed FCR and self-reported prevalent stroke. Together, these findings support FCR as an exploratory load-to-reserve indicator associated with self-reported prevalent stroke in this population-based sample. Importantly, because the analysis was cross-sectional and the outcome was self-reported prevalent stroke, these findings do not establish temporality; reverse causality remains possible because stroke may contribute to subsequent frailty, functional limitations, reduced physical activity, and lower estimated fitness.

Several additional analyses helped contextualize this association. Because FCR is a ratio-based measure, raw continuous FCR was sensitive to extreme ratio values, whereas log-transformed FCR and quartile-based FCR provided more stable estimates. Sensitivity analyses addressing low eCRF values, winsorized FCR, exclusion of extreme FCR values, and an alternative standardized FI-minus-eCRF composite showed broadly consistent positive associations. These results suggest that the observed association was not solely driven by the handling of extreme denominator-related values, while it also supported the emphasis on log-transformed and categorized representations of FCR.

The component-comparison analyses further contextualized the FCR construct. Log-transformed FCR and FCR quartiles improved apparent discrimination compared with the baseline covariate model, whereas FI-based models showed numerically higher apparent AUCs. Accordingly, FCR is best interpreted as a complementary load-to-reserve representation that contextualizes accumulated health deficits relative to estimated cardiorespiratory reserve in population-based studies.

Exploratory subgroup analyses showed directionally similar point estimates for the association between log-transformed FCR and self-reported prevalent stroke across major demographic and clinical strata, including age, sex, obesity status, hypertension, and diabetes. No statistically significant interactions were observed, suggesting no clear evidence that the association differed materially across prespecified subgroups. These subgroup findings should therefore be interpreted as exploratory and hypothesis-generating rather than confirmatory.

The rationale for FCR is grounded in the complementary information captured by FI and eCRF. FI summarizes accumulated health deficits across multiple domains and provides a scalable measure of multidimensional vulnerability, whereas eCRF provides an estimated proxy for cardiorespiratory reserve derived from routinely available demographic, anthropometric, physiological, behavioral, and lifestyle variables. Prior studies have linked higher frailty burden and lower cardiorespiratory fitness with adverse cardiovascular and cerebrovascular outcomes [18,22,25,26,27]. Within this framework, FCR summarizes the relative burden of accumulated deficits in relation to estimated cardiorespiratory reserve and may provide a compact representation of the load-to-reserve balance. This interpretation is also consistent with previous studies suggesting that frailty trajectories and functional reserve are relevant to cardiovascular and cerebrovascular health [17,28,29,30].

Several biological pathways may help explain why a higher deficit-to-reserve profile is associated with self-reported prevalent stroke. Frailty has been linked to vascular aging, impaired physiological resilience, systemic inflammation, endothelial dysfunction, and multimorbidity, all of which are relevant to cerebrovascular disease processes [31,32,33,34,35]. Chronic low-grade inflammation, often described as “inflammaging,” may contribute to endothelial injury, thrombogenic tendency, and vascular dysfunction. Multimorbidity, nutritional vulnerability, and functional limitations may further reduce resilience to ischemic or vascular stressors. In contrast, higher cardiorespiratory fitness is generally associated with more favorable cardiovascular and metabolic profiles, including better blood pressure regulation, insulin sensitivity, autonomic balance, and vascular function [36]. Thus, a higher FCR may reflect the coexistence of greater accumulated health deficits and lower estimated cardiorespiratory reserve. This profile may be related to prevalent cerebrovascular disease through overlapping pathways involving vascular aging, metabolic dysfunction, inflammation, and reduced physiological resilience. These pathways provide a biologically plausible context for the observed cross-sectional association.

This study has several methodological strengths. First, the analysis was based on NHANES 2011–2014, a nationally representative survey with standardized interviews, examinations, and complex sampling design. The analyses incorporated MEC examination weights, strata, and primary sampling units to generate survey-weighted estimates. Second, the construction of the 23-item modified FI was made transparent by providing detailed item definitions, coding rules, missing proportions, and deficit prevalence in the Supplementary Materials. Third, because FCR is a ratio-based measure, we performed multiple sensitivity analyses addressing low eCRF values, extreme FCR values, log-transformed FCR, and an alternative standardized FI-minus-eCRF composite. Fourth, we benchmarked FCR against FI, eCRF, and their joint model and further assessed variable overlap using correlation diagnostics, variance inflation factors, and overlap-aware adjustment models. These additional analyses provide context for interpreting the FCR construct relative to its individual components.

Several limitations should be considered. First, the cross-sectional design precludes assessment of temporality or causality, and reverse causality is possible because stroke may contribute to subsequent frailty, functional limitation, and reduced fitness. Accordingly, temporal and causal interpretations require longitudinal validation. Second, the outcome was based on self-reported prevalent stroke, and NHANES did not provide information on stroke onset date, subtype, severity, recurrence, or adjudicated clinical endpoints. Third, eCRF was estimated using non-exercise equations rather than measured directly by cardiopulmonary exercise testing; therefore, it should be interpreted as an estimated proxy for cardiorespiratory fitness.

Fourth, the FI used in this study was a 23-item modified FI constructed from health deficits available in NHANES 2011–2014. Although it followed the deficit accumulation framework and excluded self-reported stroke to avoid direct outcome overlap, the item set was constrained by available survey variables. Some FI items also had relatively high missing proportions, and the healthcare visits item may partly reflect healthcare access or service utilization rather than biological vulnerability alone. Fifth, as a ratio-based indicator, FCR may be influenced by small denominator values and extreme ratio distributions. We addressed this issue using log transformation, exclusion of non-positive eCRF values, winsorization, exclusion of extreme FCR values, and alternative composite analyses. Sixth, several covariates overlap conceptually with FI or eCRF construction; overlap-aware and alternative adjustment models were therefore used to examine the consistency of the association. Finally, complete-case analysis may affect the generalizability of estimates, as included and excluded eligible participants differed in several baseline characteristics (Supplementary Table S6), and residual confounding by unmeasured factors may remain. Future longitudinal studies with adjudicated incident stroke outcomes, repeated assessments of frailty and measured or estimated cardiorespiratory fitness, and external validation are required before FCR can be considered for clinical risk prediction or screening applications.

## 5. Conclusions

In this cross-sectional analysis of NHANES 2011–2014, higher FCR, particularly when modeled using log-transformed values or quartiles, was associated with greater odds of self-reported prevalent stroke among middle-aged and older adults. These findings support FCR as an interpretable exploratory load-to-reserve construct that integrates frailty burden with estimated cardiorespiratory reserve. FCR may provide a complementary framework for summarizing the relationship between accumulated health deficits and estimated cardiorespiratory reserve in population-based studies of cerebrovascular health. Further longitudinal studies with adjudicated incident stroke outcomes and external validation are needed to clarify temporality and to determine whether FCR can be considered for clinical risk prediction or screening applications.

## Figures and Tables

**Table 1:** Survey-weighted baseline characteristics of participants by FCR quartiles, based on NHANES 2011–2014.

Characteristic	Q1 (Lowest)	Q2	Q3	Q4 (Highest)	Total	*p* Value
Age, years	63.51 (0.37)	65.45 (0.26)	67.00 (0.39)	66.64 (0.43)	65.58 (0.20)	<0.001
BMI, kg/m^2^	26.41 (0.18)	28.10 (0.26)	29.36 (0.31)	34.18 (0.65)	29.36 (0.24)	<0.001
Waist circumference, cm	96.65 (0.55)	100.20 (0.60)	103.31 (0.89)	112.50 (1.13)	102.84 (0.52)	<0.001
eCRF, METs	9.40 (0.09)	8.51 (0.07)	7.48 (0.07)	6.12 (0.15)	7.95 (0.06)	<0.001
Frailty Index	0.07 (0.00)	0.16 (0.00)	0.26 (0.00)	0.43 (0.01)	0.22 (0.01)	<0.001
FCR	0.01 (0.00)	0.02 (0.00)	0.03 (0.00)	0.08 (0.01)	0.03 (0.00)	<0.001
Sex, n (weighted %)						<0.001
Male	558 (60.0%)	484 (53.1%)	397 (41.1%)	259 (27.3%)	1698 (46.1%)	
Female	320 (40.0%)	394 (46.9%)	481 (58.9%)	618 (72.7%)	1813 (53.9%)	
Race/ethnicity, n (weighted %)						0.139
Mexican American	85 (3.6%)	77 (3.8%)	70 (3.6%)	86 (4.8%)	318 (3.9%)	
Other Hispanic	98 (3.8%)	87 (3.9%)	83 (4.3%)	86 (4.5%)	354 (4.1%)	
Non-Hispanic White	359 (78.7%)	395 (77.3%)	441 (76.7%)	425 (74.3%)	1620 (76.9%)	
Non-Hispanic Black	196 (7.3%)	220 (9.0%)	212 (10.1%)	222 (11.7%)	850 (9.4%)	
Other race/multiracial	140 (6.6%)	99 (5.9%)	72 (5.2%)	58 (4.7%)	369 (5.6%)	
Education level, n (weighted %)						<0.001
Less than high school	204 (13.1%)	196 (13.6%)	269 (22.5%)	291 (25.0%)	960 (18.2%)	
High school	200 (21.0%)	203 (21.7%)	197 (21.6%)	213 (25.2%)	813 (22.3%)	
College or above	474 (66.0%)	479 (64.6%)	410 (55.8%)	373 (49.8%)	1736 (59.5%)	
Other/unknown	0 (0.0%)	0 (0.0%)	2 (0.1%)	0 (0.0%)	2 (0.0%)	
Smoking status, n (weighted %)						0.001
No	448 (52.9%)	451 (50.6%)	384 (40.9%)	395 (42.7%)	1678 (47.1%)	
Yes	430 (47.1%)	427 (49.4%)	494 (59.1%)	482 (57.3%)	1833 (52.9%)	
Alcohol consumption, n (weighted %)						0.001
No	231 (19.2%)	262 (24.1%)	278 (29.5%)	329 (31.9%)	1100 (25.9%)	
Yes	647 (80.8%)	616 (75.9%)	600 (70.5%)	548 (68.1%)	2411 (74.1%)	
Hypertension, n (weighted %)						<0.001
No	632 (73.8%)	328 (41.1%)	260 (31.5%)	161 (19.4%)	1381 (42.5%)	
Yes	246 (26.2%)	550 (58.9%)	618 (68.5%)	716 (80.6%)	2130 (57.5%)	
Diabetes mellitus, n (weighted %)						<0.001
No	834 (96.2%)	701 (85.8%)	646 (75.8%)	506 (61.1%)	2687 (80.5%)	
Yes	44 (3.8%)	177 (14.2%)	232 (24.2%)	371 (38.9%)	824 (19.5%)	
Self-reported prevalent stroke, n (weighted %)						<0.001
No	860 (97.8%)	839 (96.3%)	810 (93.3%)	750 (87.1%)	3259 (93.9%)	
Yes	18 (2.2%)	39 (3.7%)	68 (6.7%)	127 (12.9%)	252 (6.1%)	

*Notes:* Continuous variables are survey-weighted means (SEs); categorical variables are unweighted counts (weighted %). FCR is shown on the raw scale. BMI, body mass index; eCRF, estimated cardiorespiratory fitness; FCR, Frailty-to-estimated Cardiorespiratory Fitness Ratio; METs, metabolic equivalents.

**Table 2:** Survey-weighted associations of log-transformed FCR and FCR quartiles with self-reported prevalent stroke.

Exposure	Model 1 Unadjusted	Model 2 Demographic	Model 3 Socio-Lifestyle	Model 4 Clinical	Model 5 Overlap-Aware
log(FCR+0.001), per 1-SD increment	2.26 (1.86, 2.75); p<0.001	2.33 (1.90, 2.85); p<0.001	3.09 (2.32, 4.10); p<0.001	2.77 (2.13, 3.60); p<0.001	2.20 (1.82, 2.67); p<0.001
Q1	1.00 (Ref.)	1.00 (Ref.)	1.00 (Ref.)	1.00 (Ref.)	1.00 (Ref.)
Q2	1.76 (0.72, 4.27); p=0.222	1.72 (0.73, 4.06); p=0.231	1.84 (0.77, 4.36); p=0.187	1.49 (0.60, 3.71); p=0.407	1.77 (0.74, 4.27); p=0.215
Q3	3.26 (1.63, 6.54); p=0.002	3.22 (1.73, 6.02); p=0.001	3.37 (1.75, 6.46); p=0.002	2.57 (1.30, 5.09); p=0.016	3.15 (1.57, 6.34); p=0.004
Q4	6.67 (3.25, 13.69); p<0.001	7.02 (3.65, 13.48); p<0.001	8.67 (4.19, 17.94); p<0.001	6.13 (2.97, 12.66); p<0.001	6.31 (3.07, 12.97); p<0.001
*p* for trend	<0.001	<0.001	<0.001	<0.001	<0.001

*Notes:* Values are ORs (95% CIs). log(FCR+0.001) was standardized before modeling. Q1 was the reference group. CI, confidence interval; FCR, Frailty-to-estimated Cardiorespiratory Fitness Ratio; OR, odds ratio.

## Data Availability

Publicly available datasets were analyzed in this study. These data can be found in the National Health and Nutrition Examination Survey (NHANES) repository.

## Supplementary Materials

The following supporting information can be downloaded at https://www.mdpi.com/article/10.3390/bioengineering13070750/s1: Supplementary Table S1: Construction of the 23-item modified Frailty Index; Supplementary Table S2: Sensitivity analyses for FCR parameterization and extreme values; Supplementary Table S3A,B: Component-comparison and apparent model-performance analyses; Supplementary Table S4A,B: Correlation and multicollinearity diagnostics; Supplementary Table S5: Overlap-aware and alternative adjustment models; Supplementary Table S6: Baseline characteristics of included and excluded eligible participants; and Supplementary Table S7: Sensitivity analyses using alternative FI specifications.
