# Supplementary material for: Association of the Frailty-to-Estimated Cardiorespiratory Fitness Ratio with Prevalent Stroke in Middle-Aged and Older Adults: A Cross-Sectional NHANES Study
Source: Bioengineering (Basel). 2026 Jun 26;13(7):750. doi: 10.3390/bioengineering13070750 (PMC13404228; doi:10.3390/bioengineering13070750)
Supplement: Supplementary file 1 [file bioengineering-13-00750-s001.zip › bioengineering-4231734-supplementary.pdf]

Supplementary Materials

Supplementary materials for: *Association of the Frailty-to-Estimated Cardiorespiratory Fitness Ratio with Prevalent Stroke in Middle-Aged and Older Adults: A Cross-Sectional NHANES Study.*

Supplementary Table S1. Construction of the 23-item modified Frailty Index.

No.	Domain	NHANES variable	Item	Deficit coding	Missing, %	Deficit, % valid
1	Comorbidity	MCQ160A	Arthritis	1 = Yes	0.3	38.9
2	Comorbidity	MCQ160B	Congestive heart failure	1 = Yes	0.3	4.7
3	Comorbidity	MCQ160C	Coronary heart disease	1 = Yes	0.3	6.2
4	Comorbidity	MCQ160D	Angina/angina pectoris	1 = Yes	0.3	3.6
5	Comorbidity	MCQ160E	Heart attack	1 = Yes	0	6
6	Comorbidity	MCQ160L	Liver condition	1 = Yes	0.2	5.5
7	Comorbidity	MCQ160M	Thyroid problem	1 = Yes	0.3	13.9
8	Comorbidity	MCQ220	Cancer or malignancy	1 = Yes	0	14.2
9	Comorbidity	MCQ160N	Chronic bronchitis	1 = Yes	0.1	6.4
10	Comorbidity	BPQ020	Hypertension	1 = Yes	0.1	51.8
11	Comorbidity	DIQ010	Diabetes	1 = Yes	3.8	20
12	Functional limitation	PFQ061B	Difficulty standing from an armless chair	2/3/4 = any difficulty or unable	46.1	21.4
13	Functional limitation	PFQ061C	Difficulty getting in or out of bed	2/3/4 = any difficulty or unable	45.8	15.5
14	Functional limitation	PFQ061H	Difficulty stooping/crouching/kneeling	2/3/4 = any difficulty or unable	32.6	9.8
15	Functional limitation	PFQ061K	Difficulty walking between rooms	2/3/4 = any difficulty or unable	32.5	6.5
16	Functional limitation	PFQ061I	Difficulty lifting or carrying	2/3/4 = any difficulty or unable	32.5	23.2
17	Functional limitation	PFQ061J	Difficulty doing household chores	2/3/4 = any difficulty or unable	32.6	17.8
18	Functional limitation	PFQ061F	Difficulty walking for a quarter mile	2/3/4 = any difficulty or unable	34.7	23.3
19	Functional limitation	PFQ061G	Difficulty walking up 10 steps	2/3/4 = any difficulty or unable	35.3	9.4
20	Functional limitation	PFQ061D	Difficulty eating	2/3/4 = any difficulty or unable	33.5	48
21	Functional limitation	PFQ061E	Difficulty dressing	2/3/4 = any difficulty or unable	33.5	26.3
22	Healthcare use	HUQ050/HUQ051	Healthcare visits in past year	$\geq$1 healthcare visit = deficit	0	89.1
23	General health	HSD010	Fair or poor self-rated health	4/5 = fair/poor	5.3	27.3

Notes: The FI construction population included 5514 participants with complete eCRF components. Self-reported stroke was not included as an FI item. Participants were required to have at least 20 valid FI items. Missing or invalid responses included refused, don't know, and missing values unless otherwise specified. Deficit percentages are among participants with valid responses for each item.

Supplementary Table S2. Sensitivity analyses for FCR parameterization and extreme values.

**Scenario**	**N**	**Stroke events**	**Exposure**	**OR (95% CI)**	**P value**
Main: exclude eCRF raw ≤ 0	3511	252	FCR per 1-SD	1.08 (0.76, 1.53)	0.680
Main: exclude eCRF raw ≤ 0	3511	252	log(FCR + 0.001) per 1-SD	2.25 (1.83, 2.76)	< 0.001
Main: exclude eCRF raw ≤ 0	3511	252	FI z - eCRF z composite per 1-SD	1.90 (1.61, 2.25)	< 0.001
Main: exclude eCRF raw ≤ 0	3511	252	Q1	1.00 (Ref)	
Main: exclude eCRF raw ≤ 0	3511	252	Q2	1.72 (0.73, 4.06)	0.234
Main: exclude eCRF raw ≤ 0	3511	252	Q3	3.06 (1.62, 5.77)	0.003
Main: exclude eCRF raw ≤ 0	3511	252	Q4	6.46 (3.34, 12.50)	< 0.001
Main: exclude eCRF raw ≤ 0	3511	252	P for trend		< 0.001
Exclude eCRF raw < 1	3509	251	FCR per 1-SD	1.43 (1.20, 1.71)	< 0.001
Exclude eCRF raw < 1	3509	251	log(FCR + 0.001) per 1-SD	2.24 (1.84, 2.73)	< 0.001
Exclude eCRF raw < 1	3509	251	FI z - eCRF z composite per 1-SD	1.88 (1.59, 2.23)	< 0.001
Exclude eCRF raw < 1	3509	251	Q1	1.00 (Ref)	
Exclude eCRF raw < 1	3509	251	Q2	1.72 (0.73, 4.06)	0.234
Exclude eCRF raw < 1	3509	251	Q3	3.06 (1.62, 5.77)	0.003
Exclude eCRF raw < 1	3509	251	Q4	6.42 (3.32, 12.43)	< 0.001
Exclude eCRF raw < 1	3509	251	P for trend		< 0.001
Legacy floor: eCRF values < 1 set to 1	3513	252	FCR per 1-SD	1.46 (1.21, 1.75)	< 0.001
Legacy floor: eCRF values < 1 set to 1	3513	252	log(FCR + 0.001) per 1-SD	2.25 (1.85, 2.73)	< 0.001
Legacy floor: eCRF values < 1 set to 1	3513	252	FI z - eCRF z composite per 1-SD	1.90 (1.60, 2.24)	< 0.001
Legacy floor: eCRF values < 1 set to 1	3513	252	Q1	1.00 (Ref)	
Legacy floor: eCRF values < 1 set to 1	3513	252	Q2	1.72 (0.72, 4.06)	0.235
Legacy floor: eCRF values < 1 set to 1	3513	252	Q3	3.14 (1.62, 6.06)	0.003
Legacy floor: eCRF values < 1 set to 1	3513	252	Q4	6.36 (3.34, 12.12)	< 0.001
Legacy floor: eCRF values < 1 set to 1	3513	252	P for trend		< 0.001
Winsorize FCR at 99th percentile	3511	252	FCR per 1-SD	1.66 (1.46, 1.89)	< 0.001
Winsorize FCR at 99th percentile	3511	252	log(FCR + 0.001) per 1-SD	2.34 (1.88, 2.90)	< 0.001
Winsorize FCR at 99th percentile	3511	252	FI z - eCRF z composite per 1-SD	1.90 (1.61, 2.25)	< 0.001
Winsorize FCR at 99th percentile	3511	252	Q1	1.00 (Ref)	
Winsorize FCR at 99th percentile	3511	252	Q2	1.72 (0.73, 4.06)	0.234
Winsorize FCR at 99th percentile	3511	252	Q3	3.06 (1.62, 5.77)	0.003
Winsorize FCR at 99th percentile	3511	252	Q4	6.46 (3.34, 12.50)	< 0.001
Winsorize FCR at 99th percentile	3511	252	P for trend		< 0.001
Exclude top 1% FCR	3475	247	FCR per 1-SD	1.78 (1.54, 2.05)	< 0.001
Exclude top 1% FCR	3475	247	log(FCR + 0.001) per 1-SD	2.46 (1.94, 3.11)	< 0.001
Exclude top 1% FCR	3475	247	FI z - eCRF z composite per 1-SD	2.01 (1.66, 2.44)	< 0.001
Exclude top 1% FCR	3475	247	Q1	1.00 (Ref)	
Exclude top 1% FCR	3475	247	Q2	1.66 (0.69, 3.96)	0.269
Exclude top 1% FCR	3475	247	Q3	3.03 (1.59, 5.79)	0.003
Exclude top 1% FCR	3475	247	Q4	6.47 (3.30, 12.69)	< 0.001
Exclude top 1% FCR	3475	247	P for trend		< 0.001

Scenario	N	Stroke events	Exposure	OR (95% CI)	P value

Notes: ORs are from survey-weighted logistic regression models using the overlap-aware sensitivity-analysis framework unless otherwise specified. The main analysis excluded non-positive raw eCRF values and used N=3511. The legacy floor scenario retained complete-case participants by setting eCRF values below 1 to 1 and is provided only as a sensitivity comparison.

Supplementary Table S3A. Component-comparison and exploratory apparent discrimination metrics.

**Model**	**N**	**AUC**	**Delta AUC**	**Brier score**	**Discrimination slope**
Baseline	3511	0.6423	0.0000	0.0568	0.0153
Baseline + FI	3511	0.7485	0.1062	0.0545	0.0597
Baseline + eCRF	3511	0.6419	-0.0004	0.0568	0.0162
Baseline + FI + eCRF	3511	0.7495	0.1072	0.0544	0.0616
Baseline + raw FCR	3511	0.6457	0.0034	0.0568	0.0155
Baseline + log-FCR	3511	0.7305	0.0882	0.0554	0.0426
Baseline + FCR quartiles	3511	0.7205	0.0782	0.0553	0.0423

Notes: These are exploratory apparent model-performance metrics from cross-sectional survey-weighted models. The analyses were used to benchmark FCR against FI, eCRF, and their joint model.

Supplementary Table S3B. Calibration and reclassification metrics for exploratory apparent model-performance analyses.

Model	Calibration intercept (SE)	Calibration slope (SE)	IDI	Continuous NRI	Event NRI	Nonevent NRI
Baseline	0.000 (0.064)	1.000 (0.176)	0.0000	0.0000	0.0000	0.0000
Baseline + FI	0.000 (0.068)	1.000 (0.085)	0.0444	0.6372	0.1952	0.4419
Baseline + eCRF	0.000 (0.064)	1.000 (0.170)	0.0009	0.1303	-0.0330	0.1633
Baseline + FI + eCRF	0.000 (0.069)	1.000 (0.083)	0.0463	0.6116	0.1773	0.4343
Baseline + raw FCR	0.000 (0.064)	1.000 (0.167)	0.0002	0.4280	0.1139	0.3141
Baseline + log-FCR	0.000 (0.065)	1.000 (0.098)	0.0273	0.5122	0.2808	0.2314
Baseline + FCR quartiles	0.000 (0.066)	1.000 (0.103)	0.0271	0.5428	0.2386	0.3042

Notes: Calibration and reclassification indices are exploratory apparent metrics from cross-sectional models and were used to contextualize model behavior.

Supplementary Table S4A. Spearman correlation matrix for FCR components and selected overlapping covariates.

**Variable**	**FI**	**eCRF**	**FCR**	**log-FCR**	**Age**	**Female**	**BMI**	**HTN**	**DM**
FI	1.000	-0.326	0.947	0.947	0.026	0.124	0.257	0.400	0.327
eCRF	-0.326	1.000	-0.590	-0.590	-0.374	-0.522	-0.629	-0.206	-0.161
FCR	0.947	-0.590	1.000	1.000	0.143	0.261	0.421	0.409	0.327
log-FCR	0.947	-0.590	1.000	1.000	0.143	0.261	0.421	0.409	0.327
Age	0.026	-0.374	0.143	0.143	1.000	-0.012	-0.137	0.122	0.000
Female	0.124	-0.522	0.261	0.261	-0.012	1.000	0.087	0.036	-0.025
BMI	0.257	-0.629	0.421	0.421	-0.137	0.087	1.000	0.177	0.203
HTN	0.400	-0.206	0.409	0.409	0.122	0.036	0.177	1.000	0.153
DM	0.327	-0.161	0.327	0.327	0.000	-0.025	0.203	0.153	1.000

Notes: Values are Spearman correlation coefficients. HTN indicates hypertension; DM, diabetes mellitus. A focused subset of FCR components and selected overlapping covariates is shown for interpretability; the source CSV in this folder contains the full matrix.

Supplementary Table S4B. Summary of variance inflation factor diagnostics.

Model	Maximum VIF	Terms with VIF $\geq$ 5	Terms with VIF $\geq$ 10
Conventional FCR quartile	5.04	Race/ethnicity category	None
Conventional log-FCR	5.04	Race/ethnicity category	None
FI + eCRF component model	5.02	Race/ethnicity category	None
Overlap-aware log-FCR	4.98	None	None

Notes: VIF diagnostics did not indicate severe statistical collinearity for log-FCR, FCR quartiles, FI, or eCRF. Conceptual overlap between FCR components and adjustment variables remains a separate interpretive issue and was addressed through alternative adjustment models.

Supplementary Table S5. Overlap-aware and alternative adjustment models.

**Model**	**Exposure**	**Term**	**OR (95% CI)**	**P value**	**Rationale**
Unadjusted	log-FCR z-score	log-FCR z-score	2.26 (1.86, 2.75)	< 0.001	Crude association.
Unadjusted	FCR quartiles	Q4 vs Q1	6.67 (3.25, 13.69)	< 0.001	Crude association.
Demographic	log-FCR z-score	log-FCR z-score	2.33 (1.90, 2.85)	< 0.001	Demographic adjustment.
Demographic	FCR quartiles	Q4 vs Q1	7.02 (3.65, 13.48)	< 0.001	Demographic adjustment.
Conventional full	log-FCR z-score	log-FCR z-score	2.77 (2.13, 3.60)	< 0.001	Conventional clinical adjustment.
Conventional full	FCR quartiles	Q4 vs Q1	6.13 (2.97, 12.66)	< 0.001	Conventional clinical adjustment.
Overlap-aware core	log-FCR z-score	log-FCR z-score	2.20 (1.82, 2.67)	< 0.001	Overlap-aware core model.
Overlap-aware core	FCR quartiles	Q4 vs Q1	6.31 (3.07, 12.97)	< 0.001	Overlap-aware core model.
Overlap-aware plus comorbidity	log-FCR z-score	log-FCR z-score	1.98 (1.65, 2.39)	< 0.001	Overlap-aware model retaining major comorbidities.
Overlap-aware plus comorbidity	FCR quartiles	Q4 vs Q1	4.49 (2.09, 9.63)	0.001	Overlap-aware model retaining major comorbidities.
Extended adjustment including measured eCRF inputs	log-FCR z-score	log-FCR z-score	3.17 (2.31, 4.34)	< 0.001	Extended adjustment including measured eCRF inputs.
Extended adjustment including measured eCRF inputs	FCR quartiles	Q4 vs Q1	8.48 (3.89, 18.50)	< 0.001	Extended adjustment including measured eCRF inputs.

Notes: These models were used to assess whether the FCR-stroke association persisted across conventional and overlap-aware adjustment strategies. The extended adjustment model was included only as an additional sensitivity analysis to examine covariate-overlap sensitivity and was not treated as the preferred inferential model.

Supplementary Table S6. Baseline characteristics of included and excluded eligible participants.

**Characteristic**	**Type**	**Included**	**Excluded**	**P value**
Age, years	Weighted mean (SE)	65.58 (0.20)	54.73 (0.24)	< 0.001
Sex: Male	Unweighted n (weighted %)	1698 (46.1%)	1390 (47.7%)	0.335
Sex: Female	Unweighted n (weighted %)	1813 (53.9%)	1497 (52.3%)	
Race/ethnicity: Mexican American	Unweighted n (weighted %)	318 (3.9%)	328 (6.8%)	< 0.001
Race/ethnicity: Non-Hispanic Black	Unweighted n (weighted %)	850 (9.4%)	707 (11.6%)	
Race/ethnicity: Non-Hispanic White	Unweighted n (weighted %)	1620 (76.9%)	1084 (69.2%)	
Race/ethnicity: Other Hispanic	Unweighted n (weighted %)	354 (4.1%)	274 (5.0%)	
Race/ethnicity: Other race/multiracial	Unweighted n (weighted %)	369 (5.6%)	494 (7.4%)	
Education: Less than High School	Unweighted n (weighted %)	960 (18.2%)	750 (15.9%)	0.007
Education: High School	Unweighted n (weighted %)	813 (22.3%)	641 (21.2%)	
Education: College or Above	Unweighted n (weighted %)	1736 (59.5%)	1491 (62.8%)	
Education: Missing/Other	Unweighted n (weighted %)	2 (0.0%)	5 (0.1%)	
Family income-to-poverty ratio	Weighted mean (SE)	2.94 (0.08)	3.32 (0.09)	< 0.001
BMI, kg/m2	Weighted mean (SE)	29.36 (0.24)	29.24 (0.22)	0.691
Waist circumference, cm	Weighted mean (SE)	102.84 (0.52)	100.31 (0.40)	< 0.001
Pulse, bpm	Weighted mean (SE)	71.14 (0.36)	71.31 (0.31)	0.662
Self-reported stroke: No	Unweighted n (weighted %)	3259 (93.9%)	2735 (96.5%)	< 0.001
Self-reported stroke: Yes	Unweighted n (weighted %)	252 (6.1%)	152 (3.5%)	
Hypertension: No	Unweighted n (weighted %)	1381 (42.5%)	1641 (61.3%)	< 0.001
Hypertension: Yes	Unweighted n (weighted %)	2130 (57.5%)	1239 (38.4%)	
Hypertension: Missing	Unweighted n (weighted %)	0 (0.0%)	7 (0.2%)	
Diabetes: No	Unweighted n (weighted %)	2687 (80.5%)	2455 (89.3%)	< 0.001
Diabetes: Yes	Unweighted n (weighted %)	824 (19.5%)	429 (10.7%)	
Diabetes: Missing	Unweighted n (weighted %)	0 (0.0%)	3 (0.0%)	
Ever smoking: No	Unweighted n (weighted %)	1676 (47.1%)	1630 (56.1%)	< 0.001
Ever smoking: Yes	Unweighted n (weighted %)	1833 (52.9%)	1254 (43.9%)	
Ever smoking: Missing	Unweighted n (weighted %)	2 (0.0%)	3 (0.0%)	
Alcohol use: No	Unweighted n (weighted %)	1100 (25.9%)	621 (16.8%)	< 0.001
Alcohol use: Yes	Unweighted n (weighted %)	2411 (74.1%)	1490 (68.7%)	
Alcohol use: Missing	Unweighted n (weighted %)	0 (0.0%)	776 (14.5%)	

Notes: Included participants were those in the final analytical sample. Excluded participants were age-eligible participants with non-missing stroke status who were excluded because of missing eCRF components, invalid FI, missing covariates or survey design variables, or non-positive eCRF values. P values are from design-based tests.

Supplementary Table S7. Sensitivity analyses using alternative FI specifications.

**FI specification**	**Items / min valid**	**N**	**Stroke events**	**FI/FCR r**	**Overlap log-FCR**	**Overlap Q4 vs Q1**	**Clinical log-FCR**	**Clinical Q4 vs Q1**	**Rationale**
Original 23-item modified FI	23 / 20	3511	252	1.000 / 1.000	2.20 (1.82, 2.67); P=< 0.001	6.31 (3.07, 12.97); P=< 0.001	2.77 (2.13, 3.60); P=< 0.001	6.13 (2.97, 12.66); P=< 0.001	Prespecified 23-item modified FI.
Excluding hypertension/diabetes items	21 / 19	3414	229	0.970 / 0.974	2.11 (1.74, 2.57); P=< 0.001	8.79 (4.11, 18.81); P=< 0.001	2.40 (1.88, 3.07); P=< 0.001	8.82 (4.07, 19.14); P=< 0.001	Addresses hypertension/diabetes overlap with covariate adjustment.
Excluding healthcare-use item	22 / 20	3389	227	0.996 / 0.994	2.28 (1.73, 2.99); P=< 0.001	8.34 (4.11, 16.90); P=< 0.001	2.54 (1.77, 3.65); P=< 0.001	7.62 (3.57, 16.28); P=< 0.001	Addresses concern about healthcare access/use as an FI item.
Excluding functional-limitation items	13 / 12	3506	252	0.791 / 0.861	2.28 (1.80, 2.90); P=< 0.001	7.74 (3.87, 15.50); P=< 0.001	3.17 (2.02, 4.98); P=< 0.001	9.52 (3.98, 22.75); P=< 0.001	Addresses possible overlap with post-stroke functional limitation.
Comorbidity-only exploratory index	11 / 10	3506	252	0.716 / 0.800	2.22 (1.59, 3.10); P=< 0.001	7.96 (3.78, 16.78); P=< 0.001	2.19 (1.28, 3.74); P=0.011	8.23 (3.10, 21.88); P=< 0.001	Exploratory disease-burden index; not the preferred FI.

Notes: Alternative FI specifications were exploratory sensitivity analyses and were not intended to replace the prespecified 23-item modified FI. For each alternative FI, the alternative FCR was calculated as the alternative FI divided by eCRF. ORs are from survey-weighted logistic regression models; the overlap-aware model adjusted for race/ethnicity, education level, and alcohol consumption, whereas the conventional clinical model followed the main Model 4 adjustment. FI/FCR r indicates Spearman correlations of each alternative FI and alternative FCR with the original FI and FCR.
